# Reduced vertical displacement of the center of mass is not accompanied by reduced oxygen uptake during walking

**DOI:** 10.1038/s41598-017-17532-6

**Published:** 2017-12-07

**Authors:** S. R. Wurdeman, P. C. Raffalt, N. Stergiou

**Affiliations:** 1 Department of Clinical and Scientific Affairs, Hanger Clinic, 11155S, Main St., Houston, TX 77025 USA; 2 Department of Biomechanics and Center for Research in Human Movement Variability, University of Nebraska at Omaha, 6160 University Drive, Omaha, NE 68182-0860 USA; 3 Julius Wolff Institute for Biomechanics and Musculoskeletal Regeneration, Charité – Universitätsmedizin Berlin, Augustenburger Platz 1, 13353 Berlin, Germany; 4 Department of Biomedical Sciences, University of Copenhagen, Blegdamsvej 3B, 2200 Copenhagen N, Denmark; 5 College of Public Health, 984355 University of Nebraska Medical Center, Omaha, NE 68198-4355 USA

## Abstract

The six determinants of gait proposed that the goal of gait is to minimize vertical displacement of the body’s center of mass (CoM) with the objective to optimize energy expenditure. On the contrary, recent investigations suggest that reduced vertical displacement leads to an increase in energy expenditure. However, these investigations had the included subjects deliberately changing their gait, which could bias the endpoint measures. The present study investigated the effect of reduced vertical displacement of the CoM on oxygen uptake and walking economy without imposing altered gait patterns. This was accomplished by having subjects walk on a curved treadmill and on a flat treadmill. Vertical displacement of the CoM (sacrum marker displacement), oxygen uptake, walking economy, stride characteristics and lower limb joint angles were measured. There were significant differences in stride characteristics and phase dependent differences in lower limb movement pattern between the two conditions which in size were comparable to the changes observed between different speeds. The vertical displacement of the CoM was significantly reduced on the curved treadmill. This was accompanied by an increase in oxygen uptake and walking economy. These results support recent assertions that the six determinants of gait do not serve to improve walking economy.

## Introduction

In their classic work, Saunders and colleagues^1^ outlined the determinants of gait as six kinematic events occurring during natural walking including pelvis rotation, pelvic tilt, knee and hip flexion, knee and ankle interaction, and lateral pelvis displacement. These determinants purportedly serve to minimize vertical displacement of the body’s center of mass (CoM)^1^. It was suggested that minimizing the vertical displacement of the body’s CoM would result in a more energy efficient gait. However, at the time of their conception, little evidence beyond anecdotal observation was provided to support this suggestion. Nevertheless, the ideas of Saunders and colleagues^1^ went relatively unchallenged for forty years until the last two decades where the functional role of the six determinants in minimizing the vertical displacement of the CoM has been questioned in a few studies^2–6^.

Furthermore, the general notion that minimizing vertical displacement of the CoM results in improved energetic efficiency has been questioned in several studies. Ortega and Farley^7^ had subjects adjusting their gait under the influence of visual feedback with the objective of walking with minimal vertical motion of their CoM. The subjects altered their gait pattern by increasing hip, knee and ankle flexion which effectively reduced the vertical displacement of the CoM. Contrary to what could be expected based on the theory by Saunders and colleagues^1^, this altered gait strategy increased the metabolic cost significantly^7^. In an extension of these observations, Gordon *et al*.^8^ instructed subjects to change their stride length and thereby the vertical displacement of the CoM. While the CoM displacement increased with increase in stride length, the cost of transport was at its minimum during walking with the preferred stride length^8^. In a related study, Massaad and colleagues^9^ also used visual feedback to reduce vertical displacement, but then added an increased vertical displacement scenario by having a “bouncy condition” in which subjects were asked to deliberately over-exaggerate the distance their CoM rises and falls during walking. Increased oxygen consumption was observed during walking with both reduced and increased vertical CoM displacement^9^.

However, one crucial aspect of all the above mentioned studies though was that they forced the subject to deliberately change their gait pattern to achieve a decreased or exaggerated vertical displacement of the CoM. As a consequence, the novel (atypical) gait strategy due to altered gait mechanics and muscle activity could have caused the observed increased energy consumption which cannot thus be credited solely to a decreased vertical displacement of the CoM. Thus, we believe that the original hypothesis by Saunders and colleagues^1^ who suggested that reduced vertical displacement of the CoM results in reduced oxygen uptake remains to be tested through an innovative experimental set-up that will utilize a natural gait pattern.

**Figure 1:**
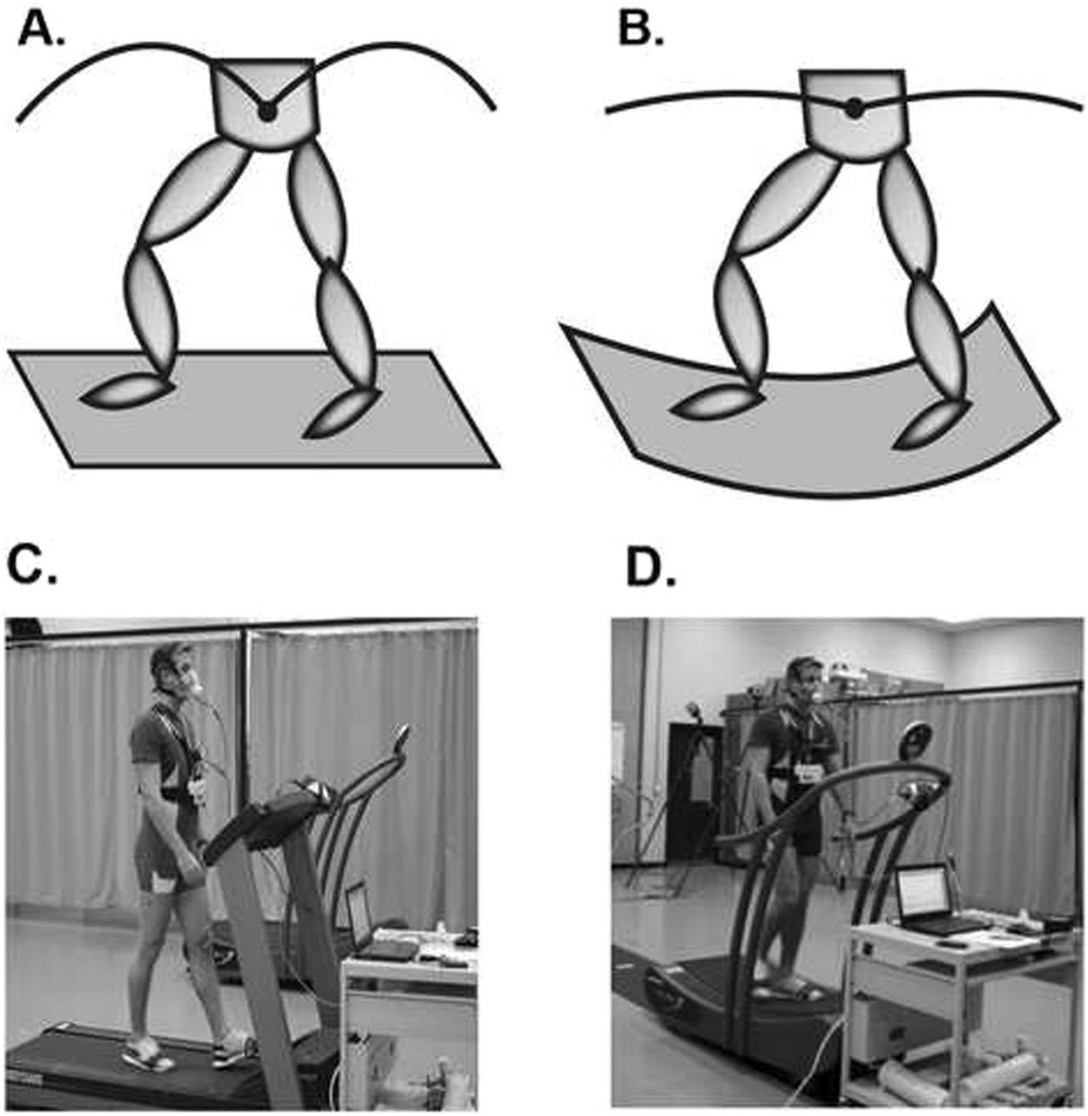
Illustration of changes in body’s CoM motion. During typical walking (**A**), the body’s CoM has an arc-like motion. However, because a curved treadmill lowers the CoM during single support when the foot is in the lowest point of the curved path, the curved treadmill flattens the CoM trajectory (**B**). Subject walking on the flat (**C**) and curved (**D**) treadmill while oxygen consumption and CoM vertical displacement is being captured.

Therefore, in the present study we wanted to challenge the general notion that minimizing vertical displacement of the CoM would result in improved energetic efficiency without imploring a deliberately unnatural gait pattern. This was accomplished by using a treadmill with a curved platform, where it is possible to translate the arc motion that the CoM typically follows, to an arc motion below the feet. It could be expected that such a translation of motion to the feet would minimize the vertical displacement occurring at the more superior region of the CoM (Fig. 1). The foot makes contact with an elevated part of the curve rather than falling (and thus lowering the CoM) to the ground, thereby decreasing vertical displacement.

Through this novel experimental set-up we sought to determine the effect of reduced vertical displacement of the CoM on the oxygen uptake and walking economy without deliberately altering the natural gait pattern. We utilized two treadmills, curved and flat, to manipulate the vertical displacement of the body’s CoM without having individuals consciously alter their gait. We also utilized different walking speeds (0.67 m/s, 1.12 m/s and 1.56 m/s) to enhance the generalizability of our results by identifying the effect that speed may have on our outcomes.

Based on the original idea of Saunders and colleagues, we hypothesized that a reduced vertical displacement of the body’s CoM will reduce oxygen uptake and improve walking economy. We also hypothesized that using our experimental set-up, subjects had a natural gait pattern. We sought to answer this hypothesis by examining the movement characteristics of the lower limb.

## Methods

In contrast to a regular flat treadmill, the curved treadmill of the present study is non-motorized. This difference could bias the results. Therefore, the primary study was supplemented by a secondary study in which five subjects returned to the lab to walk on a motorized curved treadmill and a traditional motorized flat treadmill. The purpose of this secondary study was to investigate the difference in vertical displacement, oxygen uptake, stride characteristics, and joint angles during walking on a motorized flat treadmill and a motorized curved treadmill. Thus, if differences were observed by the primary study, the results of the secondary study would tease out potential bias of the curved treadmill being non-motorized. In the following, the methodology and results of the primary study are presented and discussed while the details of the secondary study are presented in the supplementary material.

### Subjects

Inclusion criteria included subject age between 19–35 years, routinely exercise for at least three hours a week and no history of neuromuscular or musculoskeletal injuries or diseases which could affect gait. The ten subjects (7 male, 3 female) included had a mean (SD) age of 21.3 (2.67) years, body mass of 75.38 (14.64) kg and height of 180.78 (6.77) cm. All participants were informed of the experimental conditions and gave their written consent to participate in the study and to the publication of identifying images. The study was approved by the by the Institutional Review Board of the University of Nebraska Medical Center, and the study was carried out in accordance with the approved guidelines.

### Experimental protocol and measurements

Upon arrival to the laboratory, a total of 27 reflective markers were placed on anatomic landmarks of the subjects. Bilaterally: 1) anterior superior iliac spines, 2) posterior superior iliac spines, 3) greater trochanters, 4) midlateral thighs, 5) lower front thighs, 6) lateral knees, 7) tibial tubercles, 8) lower lateral shanks, 9) lateral ankles, 10) top of the second metatarsophalangeal (MTP) joints, 11) posterior heels, 12) lateral fifth MTPs, and 13) lateral calcanei. An additional single marker was placed on the sacrum. Finally, the subjects were fitted with an oxygen consumption (VO_2_) mask.

The subject walked at three different speeds (0.67, 1.12 and 1.56 m/s) on both a standard flat treadmill (T280S, Bodyguard Fitness, QC, Canada) and a non-motorized curved treadmill (Curve, Woodway USA Inc., Waukesha, WI USA,) in randomized order (see Fig. 1). Each walking trial consisted of 1–2 minutes of speed familiarization during which the subjects reached steady state in oxygen uptake, immediately followed by 3 minutes of steady state walking. During each walking trial three dimensional marker trajectories were recorded using a 12 camera motion capture system operating at 60 Hz (Motion Analysis Corp., Santa Rosa, CA). VO_2_ was continuously measured using an online COSMED breath-by-breath system and averaged for every 5 second (COSMED K4b^2^, Chicago, IL, USA).

### Analysis

The oxygen uptake and kinematic data recorded during each walking trial were analyzed in a similar manner described below.

### Stride characteristics

Toe off and heel strike events of right and left leg were identified using the coordinate-based method suggested by Zeni and colleagues^10^. Toe off was identified as a local minimum in the x-coordinate data (anterior-posterior direction) of the toe marker indicating a change from a backward motion during the contact phase to a forward motion during the initial part of the swing phase. Heel strike was identified as a local maximum in the x-coordinate data of the heel marker indicating a change from a forward motion during the end of the swing phase to a backward motion during the contact phase. From these events, stride time (the time from heel strike on one leg to the subsequent heel strike on the same leg), contact time and swing time were calculated for each stride on the right and left leg. An average of each parameter was calculated based on 90 strides extracted from each trial from each subject.

### Vertical CoM trajectories

The present study used the vertical movement of the sacrum marker in the global coordinate system as a surrogate of the CoM vertical displacement^11^. The range of CoM vertical displacement of each stride in millimeters was normalized to the leg length. Leg length was calculated as the average of the vertical distance between greater trochanter and the ankle on both legs during static upright stance. The range of CoM vertical displacement from each stride was averaged across all 90 strides.

### Joint angles

Right hip, knee and ankle joint angles were calculated using a modified Helen Hayes marker setup algorithm^12^ and time normalized to 100% of each stride. An ensemble average joint angle curve was calculated across all strides for each joint. In addition, the range of joint movement was calculated for each stride and averaged across all 90 strides.

### VO_2_ and walking economy

VO_2_ measured as liter oxygen per minute was averaged across the three minutes walking trial and normalized to the body mass of the subject (ml O_2_/min/kg). Subsequently, VO_2_ was normalized to the walking speed (ml/kg/m) to provide a measure of cost of movement comparable at different speeds as done by Foster and Lucia^13^.

### Statistics

Coordinate data set from subject 9 was incomplete and only CoM displacement and oxygen uptake data from this subject is included in the statistical analysis. Thus, n = 10 for CoM displacement and oxygen uptake data and n = 9 for stride characteristics and joint angles data.

A two-way ANOVA for repeated measures was applied to investigate the effect of walking condition (curved vs. flat treadmill) and walking speed (independent factors) and the interaction of condition and speed on the dependent variables of stride time, contact time, swing time, range of joint movement, vertical CoM displacement, oxygen uptake and walking economy. Statistical power and effect size (eta squared: η^2^) was reported for each independent factor and interaction. Effect sizes between 0.20–0.33 was considered small, between 0.33–0.50 was considered medium and above 0.5 was considered high^14,15^. In the case of a significant effect, a Holm-Sidak post hoc test was applied to evaluate the between speed and between condition differences. Level of significance was set at 0.05. All scalar statistics were performed in SigmaPlot (version 13.0.83; Systat Software, Inc. 2014, Erkrath, Germany).

Differences in the joint angle trajectories between walking conditions and walking speeds were investigated using statistical parametric mapping (SPM). The fundamental principles of SPM are described in details elsewhere^16–18^. Briefly, each joint angle trajectories (54 = 9 subjects × 2 conditions × 3 speeds) are regarded as a single vector field **r**(*q*)* = *{*rx*(*q*) *ry*(*q*)}, where *q* represents time. By applying a two-way ANOVA with interaction (walking condition and speed), three *F* statistics were computed separately at each time point *q*. Random field theory’s analytical descriptions of smooth Gaussian field behavior was used to calculate the critical threshold F* that identically smooth Gaussian fields would reach in only 5% of identical, repeated experiments. Thus, *F* trajectories which exceed F* would indicate a significant effect of the independent factors or an interaction^19^. All SPM calculations were performed in Matlab using the scripts provided at www.spm1d.org.

To estimate the size of any walking condition effect, the average absolute difference between the mean joint angles of the two treadmills was calculated for each joint and averaged across all subjects. To estimate the size of any speed effect, the average absolute difference between the mean joint angles at 0.67 m/s and 1.12 m/s and between 1.12 m/s and 1.56 m/s for each joint was calculated and averaged across all subjects.

## Results

### Vertical CoM displacement

**Figure 2:**
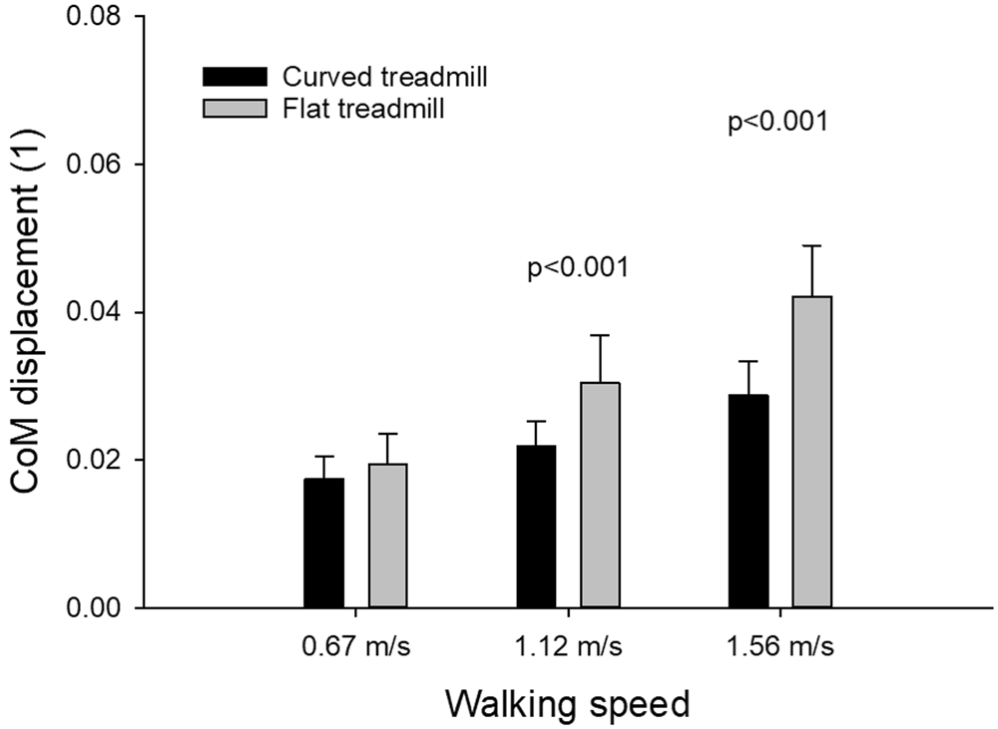
Vertical displacement of CoM. Mean and SD CoM vertical displacement (unit less) during curved and flat treadmill walking at 0.67 m/s, 1.12 m/s and 1.56 m/s.

There was overall effect of speed (F(2,18) = 174.3, p < 0.001, power = 100%, η^2^ = 0.95) and treadmill type (F(1,9) = 47.8, p < 0.001, power = 100%, η^2^ = 0.84) and an interaction effect (F(2,18) = 10.3, p = 0.001, power = 95%, η^2^ = 0.53) on the vertical CoM displacement (Fig. 2). With each increment in walking speed the displacement increased significantly for both treadmills (p ≤ 0.007 for the curved treadmill and p < 0.001 for the flat treadmill). There was a significant difference in the vertical displacement between the two treadmills at 1.12 m/s (p < 0.001) and 1.56 m/s (p < 0.001).

### Oxygen uptake and walking economy

**Figure 3:**
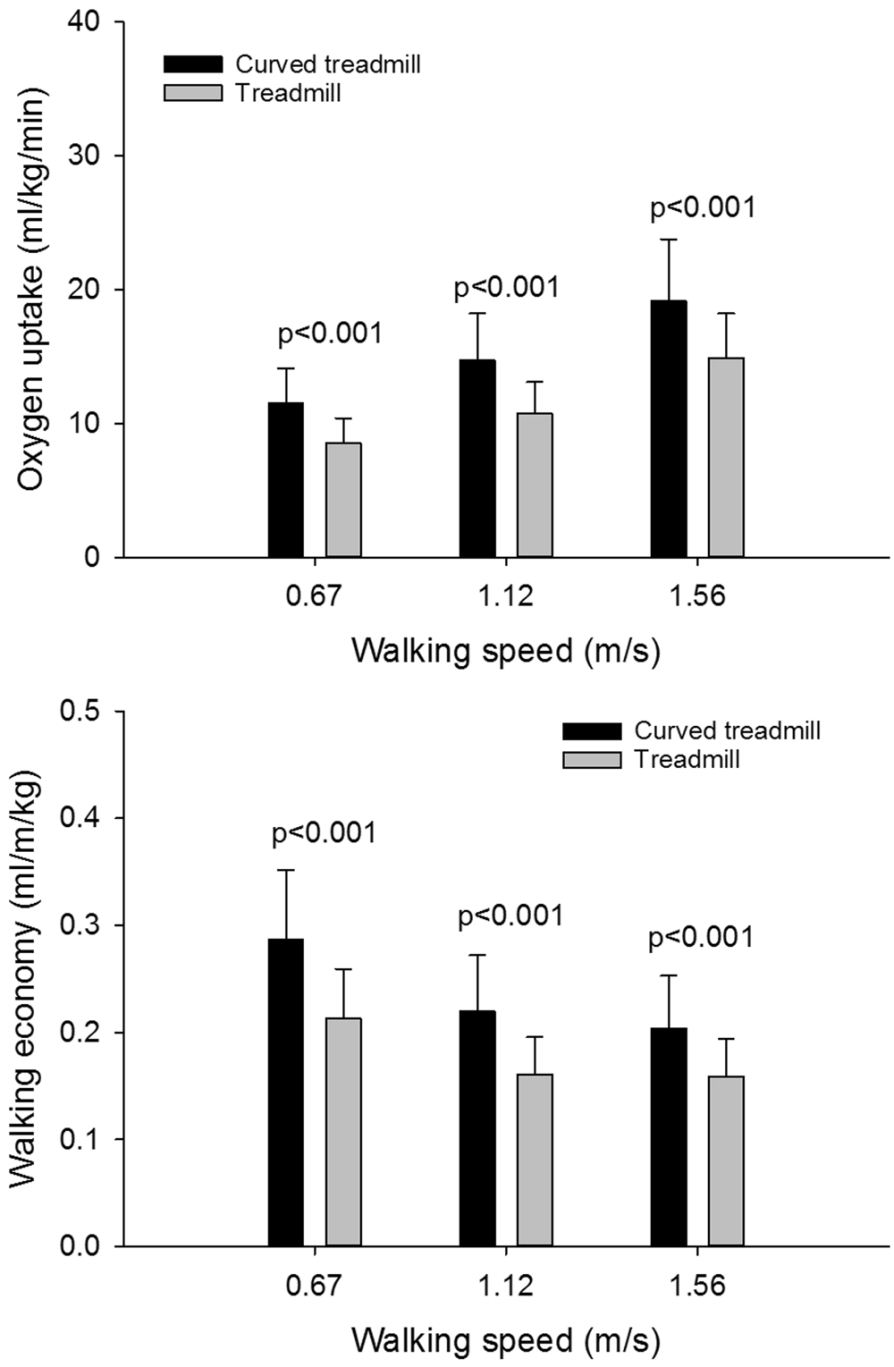
Oxygen uptake and walking economy. Mean and SD oxygen uptake (top graph) and walking economy (bottom graph) during curved and flat treadmill walking at 0.67 m/s, 1.12 m/s and 1.56 m/s.

There was an overall effect of both walking speed (F(2,18) = 98.6, p < 0.001, power = 100%, η^2^ = 0.92) and treadmill type (F(1,9) = 61.1, p < 0.001, 001, power = 100%, η^2^ = 0.87) and significant effect of the interaction of speed and treadmill (F(2,18) = 6.1, p = 0.01, power = 75%, η^2^ = 0.40) on the oxygen uptake (Fig. 3). The post-hoc test revealed that the oxygen uptake was significantly higher during curved treadmill walking compared to the flat treadmill walking at all speeds (p < 0.001 in all cases). The oxygen uptake increased significantly with increase in walking speed (p < 0.001 in all cases) for both treadmills (Fig. 3).

There was an overall effect of both walking speed (F(2,18) = 67.9, p < 0.001, power = 100%, η^2^ = 0.88) and treadmill type (F(1,9) = 70.0, p < 0.001, power = 100%, η^2^ = 0.89) and a significant effect of the interaction of treadmill and speed (F(2,18) = 17.4, p < 0.001, power = 100%, η^2^ = 0.66) on the walking economy (Fig. 3). The walking economy was significantly higher during curved treadmill walking compared to the flat treadmill walking at all speeds (p < 0.001 in all cases). For the curved treadmill the walking economy decreased significantly with each increase in walking speed (p < 0.001 for 0.67 m/s to 1.12 m/s and p = 0.041 for 1.12 m/s to 1.56 m/s). For the flat treadmill, the walking economy decreased significantly (p < 0.001) from 0.67 m/s to 1.12 m/s but no change was observed from 1.12 m/s to 1.56 m/s (Fig. 3).

### Stride characteristics

There was an overall effect of walking speed for all three variables on both the right and left leg (right stride time: F(2,16) = 355.1, p < 0.001, power = 100%, η^2^ = 0.90; left stride time: F(2,16) = 353.9, p < 0.001, power = 100%, η^2^ = 0.90; right contact time: F(2,16) = 428.5, p < 0.001, power = 100%, η^2^ = 0.98; left contact time: F(2,16) = 404.6, p < 0.001, power = 100%, η^2^ = 0.98; right swing time: F(2,16) = 146.9, p < 0.001, power = 100%, η^2^ = 0.95; left swing time: F(2,16) = 162.9, p < 0.001, power = 100%, η^2^ = 0.95; Table 1). Stride time, contact time and swing time decreased with each increase in walking speed for both treadmills (p < 0.001 in all cases). There was a significant interaction effect of walking condition on stride time (right stride time: F(2,16) = 7.6, p = 0.005, power = 85%, η^2^ = 0.49; left stride time: F(2,16) = 7.6, p = 0.005, power = 85%, η^2^ = 0.49) and on contact time (right contact time: F(2,16) = 9.2, p = 0.002, power = 92%, η^2^ = 0.54; left contact time: F(2,16) = 9.9, p = 0.002, power = 94%, η^2^ = 0.55). In the post hoc tests revealed that the stride time and contact time at 1.12 m/s and 1.56 m/s on the curved treadmill were significantly lower compared to the flat treadmill (p < 0.01 in all cases) (Table 1).

**Table 1:** Walking stride characteristics.

Speed	Curved treadmill			Flat treadmill		
	0.67 m/s	1.12 m/s	1.56 m/s	0.67 m/s	1.12 m/s	1.56 m/s
Right ST (s)	1.54 ± 0.14	1.26 ± 0.08^a^	1.09 ± 0.04^a,b^	1.54 ± 0.06	1.18 ± 0.05^a,c^	1.01 ± 0.03^a,b,c^
Left ST (s)	1.54 ± 0.14	1.26 ± 0.08^a^	1.09 ± 0.04^a,b^	1.54 ± 0.06	1.18 ± 0.05^a,c^	1.01 ± 0.03^a,b,c^
Right CT (s)	1.02 ± 0.10	0.79 ± 0.05^a^	0.66 ± 0.03^a,b^	1.03 ± 0.05	0.73 ± 0.04^a,c^	0.60 ± 0.02^a,b,c^
Left CT (s)	1.02 ± 0.10	0.78 ± 0.04^a^	0.66 ± 0.03^a,b^	1.02 ± 0.06	0.74 ± 0.06^a,c^	0.61 ± 0.04^a,b,c^
Right SWT (s)	0.52 ± 0.05	0.47 ± 0.02^a^	0.43 ± 0.01^a,b^	0.51 ± 0.02	0.44 ± 0.02^a^	0.41 ± 0.01^a,b^
Left SWT (s)	0.52 ± 0.04	0.46 ± 0.02^a^	0.43 ± 0.01^a,b^	0.52 ± 0.02	0.44 ± 0.02^a^	0.41 ± 0.01^a,b^

Stride characteristics (mean ± SD) during curved and flat treadmill walking at 0.67 m/s, 1.12 m/s and 1.56 m/s.

ST: stride time, CT: contact time, SWT: swing time, a: significantly different compared to 0.67 m/s on the same treadmill, b: significantly different compared to 1.12 m/s on the same treadmill, c: significantly different from the curved treadmill at the same walking speed.

### Joint angles

There was a significant overall effect of walking condition (F(1,8) = 99.0, p < 0.001, power = 100%, η^2^ = 0.93), walking speed (F(2,16) = 642.3, p < 0.001, power = 100%, η^2^ = 0.99) and the interaction (F(2,16) = 7.7, p = 0.004, power = 86%, η^2^ = 0.49) on the hip range of motion (Table 2). The hip range of motion increased significantly with each speed increment for both treadmills (p < 0.001 in all cases). At each walking speed the hip range of motion was higher on the curved treadmill compared to the flat treadmill (p < 0.001 in all cases). There was a significant overall effect of walking condition (F(1,8) = 112.6, p < 0.001, power = 100%, η^2^ = 0.93), walking speed (F(2,16) = 10.3, p = 0.001, power = 95%, η^2^ = 0.56) and the interaction (F(2,16) = 64.3, p < 0.001, power = 100%, η^2^ = 0.89) on the knee range of motion (Table 2). During curved treadmill walking, the knee range of motion increased significantly (p < 0.001) from 0.67 m/s to 1.12 m/s but not from 1.12 m/s to 1.56 m/s. During flat treadmill walking, the knee range of motion was significantly lower at 1.56 m/s compared to the lower speeds. At 1.12 m/s and 1.56 m/s, the knee range of motion was significantly lower on the flat treadmill compared to the curved treadmill (p < 0.001 in both cases). There was a significant overall effect of speed (F(2,16) = 21.0, p < 0.001, power = 100%, η^2^ = 0.72) and the interaction (F(2,16) = 5.3, p < 0.017, power = 66%, η^2^ = 0.40) on the ankle range of motion (Table 2). On both treadmills the ankle range of motion increased from 0.67 m/s to 1.12 m/s (p ≤ 0.004 in both cases) with no further increase.

**Table 2:** Joint range of motion.

Speed	Curved treadmill			Flat treadmill		
	0.67 m/s	1.12 m/s	1.56 m/s	0.67 m/s	1.12 m/s	1.56 m/s
Hip (°)	32.3 ± 3.6	37.5 ± 3.7^a^	42.3 ± 3.7^a,b^	27.7 ± 3.2^c^	31.3 ± 2.9^a,c^	35.4 ± 2.9^a,b,c^
Knee (°)	58.4 ± 4.9	64.9 ± 3.8^a^	66.9 ± 3.6^a^	57.8 ± 4.5	59.6 ± 2.8^c^	55.5 ± 3.3^b,c^
Ankle (°)	19.7 ± 4.0	24.0 ± 2.5^a^	25.0 ± 2.5^a^	21.4 ± 3.1	24.2 ± 1.3^a^	24.0 ± 1.6^a^

Range of motion (mean ± SD) of the right hip, knee and ankle joint angle during curved and flat treadmill walking at 0.67 m/s, 1.12 m/s and 1.56 m/s.

a: significantly different compared to 0.67 m/s on the same treadmill, b: significantly different compared to 1.12 m/s on the same treadmill, c: significantly different from the curved treadmill at the same walking speed.

**Figure 4:**
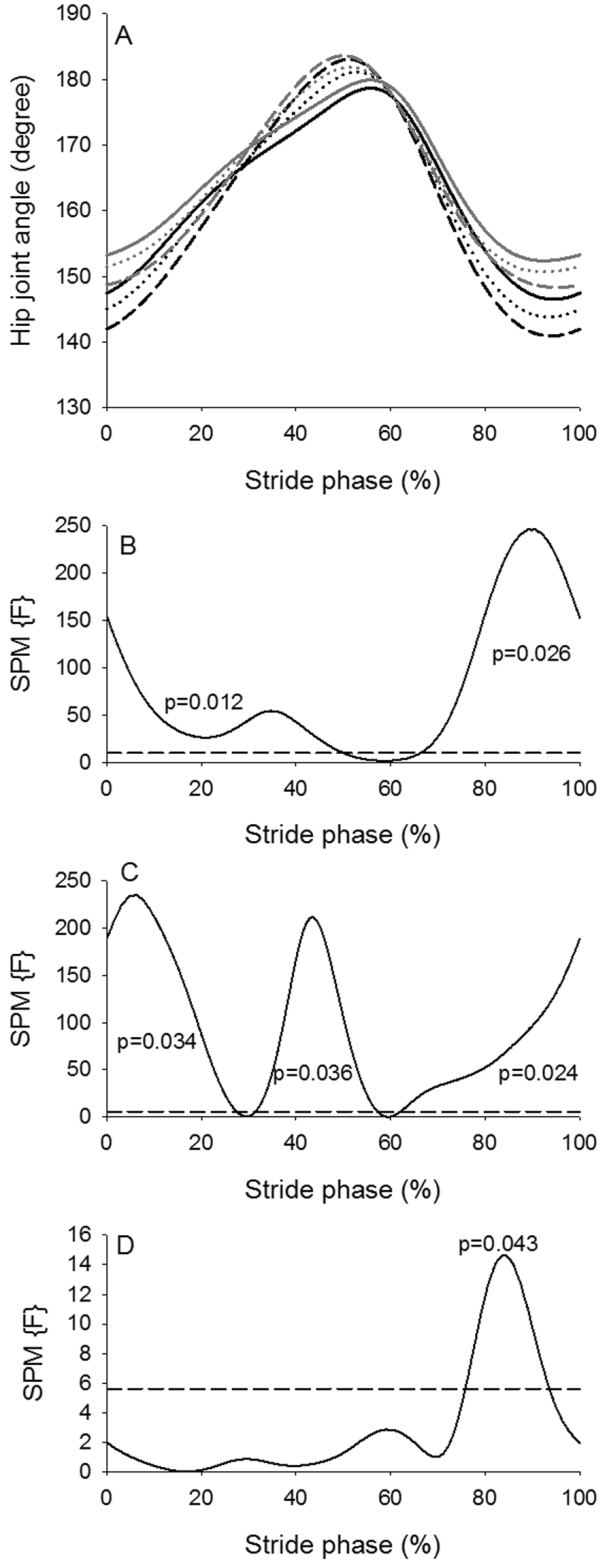
Hip joint angle. (**A**) Hip joint angle across the stride phase (hip angle of 180° equals anatomic position) for the curved treadmill (black lines) and flat treadmill (gray lines) at 0.67 m/s (solid lines), 1.12 m/s (short dashed lines) and 1.56 m/s (long dashed lines). (**B**–**D**) SPM trajectory (solid lines) across the stride phase for treadmill (**B**), speed (**C**) and interaction (**D**) effects. The dashed line indicates level of significance of 5%. SPM trajectory above the dashed line indicates a significant effect of the hip angle.

**Figure 5:**
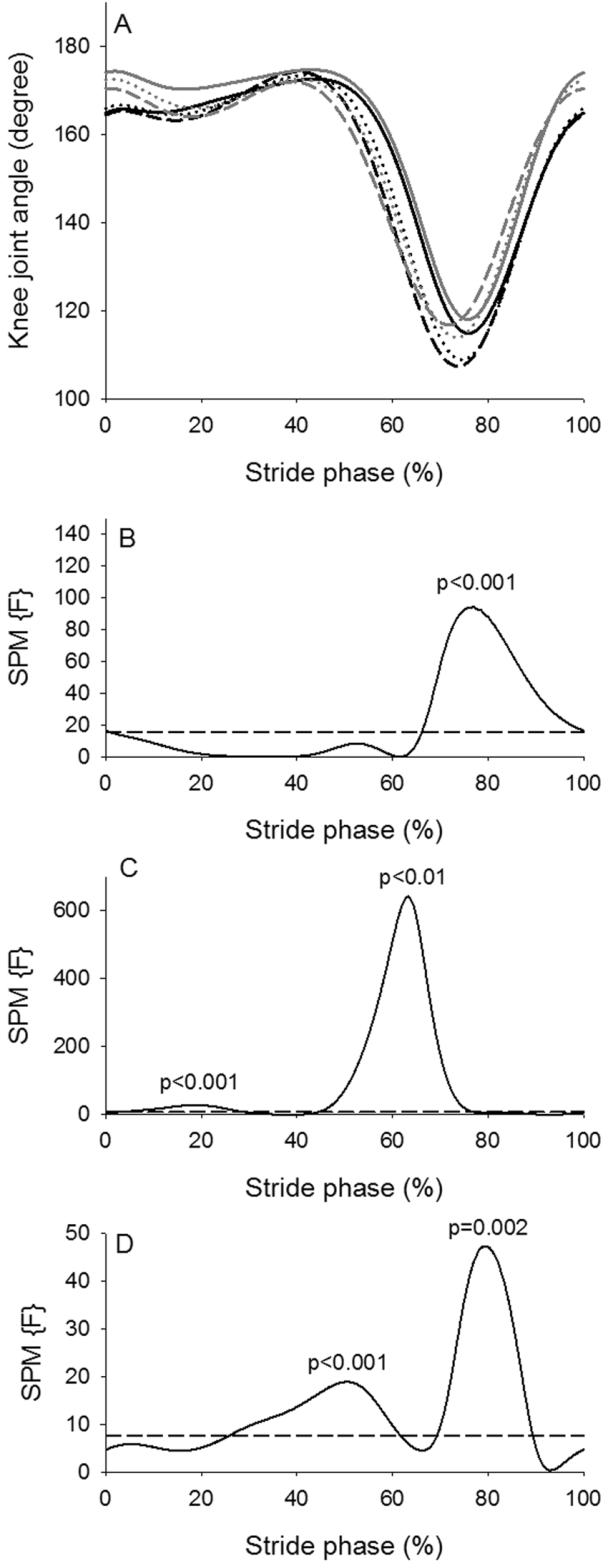
Knee joint angle. (**A**) Knee joint angle across the stride phase (knee angle of 180° equals anatomic position) for the curved treadmill (black lines) and flat treadmill (gray lines) at 0.67 m/s (solid lines), 1.12 m/s (short dashed lines) and 1.56 m/s (long dashed lines). (**B**–**D**) SPM trajectory (solid lines) across the stride phase for treadmill (**B**), speed (**C**) and interaction (**D**) effects. The dashed line indicates level of significance of 5%. SPM trajectory above the dashed line indicates a significant effect of the knee angle.

**Figure 6:**
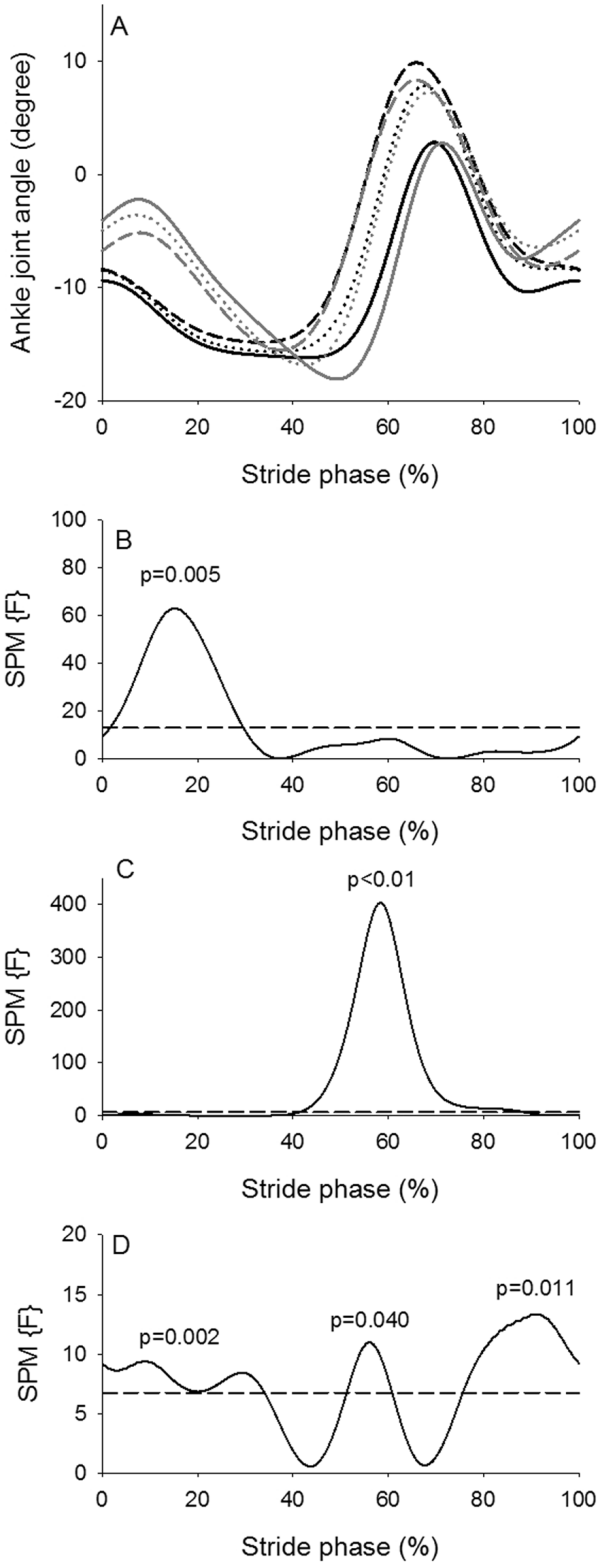
Ankle joint angle. (**A**) Ankle joint angle across the stride phase (ankle angle of 0° equals anatomic position) for the curved treadmill (black lines) and flat treadmill (gray lines) at 0.67 m/s (solid lines), 1.12 m/s (short dashed lines) and 1.56 m/s (long dashed lines). (**B**–**D**) SPM trajectory (solid lines) across the stride phase for treadmill (**B**), speed (**C**) and interaction (**D**) effects. The dashed line indicates level of significance of 5%. SPM trajectory above the dashed line indicates a significant effect of the ankle angle.

The SPM analysis of the hip angle trajectories (Fig. 4A) revealed an overall significant effect of walking condition (Fig. 4B) during most of the stride phase (approximately 0–50% and 65–100%). There was in general more hip flexion during these parts of the stride phase during curved treadmill walking (Fig. 4B). The difference in hip joint angle between the two treadmills was approximately 3 degrees at all walking speeds (Table 3). With increase in walking speed the hip flexion increased during the first (approximately 0–25%) and last part (approximately 60–100%) of the stride phase and the hip extension increased during the middle part (approximately 30–55%) of the stride phase (Fig. 4C). The size of the effect of walking speed on hip joint angle was between 1.83 and 2.38 degrees (Table 4). The interaction indicated that the hip flexion during the last part of the stride phase at 0.67 m/s (approximately 75–95%) was greater compared to 1.56 m/s at the flat treadmill (Fig. 4D). There was a significant effect of walking condition on the knee joint trajectory during the last part of the stride phase (approximately 65–100%) (Fig. 5B). There was in general a greater knee flexion during curved treadmill walking compared to flat treadmill walking (Fig. 5A). The difference in knee joint angle between the two treadmills was between 4.52 and 5.76 degrees at all walking speeds (Table 3). There was significant speed effect on knee flexion at mid-stance (approximately 10–25%) and at late stance to early swing phase (approximately 45–75% stride phase) with greater knee flexion at higher speeds (Fig. 5C). The size of the effect of walking speed on knee joint angle was between 2.55 and 5.19 degrees (Table 4). There was an interaction effect from approximately 25–60% and 70–90% of stride phase, where the knee flexion at the lowest speed for both treadmills were significantly less compared to the higher speeds (Fig. 5D). There was a significant effect of walking condition on the ankle trajectory during the first part of the stance phase (approximately 0–30% of stride phase) (Fig. 6B). Curved treadmill walking was characterized by a greater dorsiflexion compared to flat treadmill walking (Fig. 6A). The difference in ankle angle between the two treadmills was between 3.04 and 4.10 degrees at all walking speeds (Table 3).There was a significant effect of walking speed between 40 to 80% of stride phase, where a greater plantar flexion was observed at higher speeds (Fig. 6C). The differences in ankle angle with increase in walking speed were between 2.13 and 2.92 degrees (Table 4). Significant interaction between condition and speed was observed through large part of the stride phase indicating that the ankle motion differed between low walking speeds for one treadmill and high walking speed for the other treadmill and vice versa (Fig. 6D).

**Table 3:** Between-treadmill differences in joint angles.

Walking speed (m/s)	0.67 m/s	1.12 m/s	1.56 m/s
Hip angle difference (degree)	3.07 ± 0.9	3.10 ± 1.0	3.30 ± 1.1
Knee angle difference (degree)	4.62 ± 2.3	4.52 ± 1.8	5.76 ± 1.2
Ankle angle difference (degree)	4.10 ± 1.6	3.07 ± 1.4	3.04 ± 1.0

Mean enlsemble difference in the right hip, knee and ankle joint angle (mean ± SD) between curved and flat treadmill walking at 0.67 m/s, 1.12 m/s and 1.56 m/s.

**Table 4:** Between-speed differences in joint angles.

Speed (m/s)	Curved treadmill		Flat treadmill	
	0.67–1.12	1.12–1.56	0.67–1.12	1.12–1.56
Hip angle difference (degree)	2.38 ± 0.4	2.03 ± 0.4	2.17 ± 0.6	1.83 ± 0.4
Knee angle difference (degree)	3.34 ± 0.7	2.55 ± 1.0	5.19 ± 2.4	2.72 ± 0.4
Ankle angle difference (degree)	2.53 ± 0.8	2.13 ± 1.8	2.92 ± 0.8	2.18 ± 0.7

Mean ensemble difference in the right hip, knee and ankle joint angle (mean ± SD) between 0.67 m/s and 1.12 and between 1.12 m/s and 1.56 m/s for the curved and flat treadmill.

## Discussion

The present study investigated if minimizing vertical displacement of the CoM would result in improved energetic efficiency during walking. A curved formed treadmill was used to translate the typical arc motion of the CoM during walking to the feet. This translated motion was expected to minimize the vertical displacement without a deliberate alteration of the gait pattern. Based on the original idea of Saunders and colleagues, we hypothesized that the reduction in the vertical displacement of the CoM would reduce oxygen uptake and improve walking economy. This hypothesis could not be confirmed. In contrast, we observed an increase in both oxygen uptake and an impaired walking economy. Furthermore, while we observed a different lower limb movement pattern during curved treadmill walking compared to that during flat treadmill walking, the differences were relative small.

The results of the present study support the observations from previous studies^7–9^ that reduction in vertical displacement of CoM is not accompanied by a reduction in oxygen uptake or walking economy. This, combined with the observations of increased energy cost during walking with over-exaggerated CoM displacement made by Massaad and colleagues, suggests that an optimal amount of CoM displacement exists that does not constitute a maximum or minimum. While this appears to be contradictory to the theory of Saunders and colleagues^1^, it appears to be in agreement with an alternative theory proposed by Cavagna and colleagues^20,21^ suggesting that the stance leg acts like an inverted pendulum during walking. In this theory, the exchange between potential and kinetic energy during each stride requires a certain amount of vertical lift of the CoM^22^. It could be speculated that an intermediate amount of vertical displacement is optimal for the passive exchange between energy and minimizes the energy expenditure during walking. During walking, the metabolic cost is primarily related to the vertical and horizontal translation of the CoM and the swinging motion of lower limbs. The latter factor has been suggested to account for only a minor part of the total energy consumption due the use of passive structures^23,24^. In contrast, the two first factors have been considered to account for a considerable contribution. Thus, the vertical support of the CoM has been shown to reflect approximately 50% of the metabolic cost^25^ and the horizontal translation to reflect approximately 47%^26^. The study by Gottschall and Kram (2003) observed a linear relationship between applied impeding horizontal force to the waist and the subsequent increases in the metabolic cost, but a curvilinear relationship between applied aiding force and the subsequent decrease in metabolic cost during walking^26^. In light of the inverted pendulum theory by Cavagna and colleagues^20,21^, this could suggest that the unassisted walking condition constitutes an optimal interaction of the mechanical degrees of freedom enabling periodic energy exchange which is ‘disturbed’ differently when subjected to an external force (impeding or aiding).

While the theory of the six determinants of gait by Saunders and colleagues^1^ has been widely accepted during the second half of the 20^th^ century and featured in clinical and scientific textbooks^27,28^, previous studies as well as the present study question the validity of both the functional role of the six determinants in reducing the vertical displacement of the CoM^2–6,29^ and the notion that reduced displacement would lower the energy cost^7–9^. An alternative interpretation of the six determinants were offered by Kuo^22^, who suggested that they should be referred to as *‘six kinematic features of gait’* to which the mechanistic reasons for such remain unsolved.

The present study further compared the gait pattern when walking on a curved and flat treadmill walking by means of stride characteristics and joint angle trajectories. At the two highest walking speeds, the stride time increased on the curved treadmill compared to the flat treadmill through an increase in contact time. This could be due to an earlier initiated arc motion of the foot during ground contact on the curved treadmill compared to the horizontal motion of the foot during the ground contact on the flat treadmill. The range of motion analysis and joint angle trajectory analysis both indicated that significant differences were mostly observed for the hip joint. The hip joint range of motion was in general increased on the curved treadmill primarily through increased hip flexion at heel strike. This could indicate that the leg is moved further in front of the body to seek contact with the curved treadmill belt compared to the flat treadmill. Alternatively, it is possible that subjects were subconsciously trying to “climb” up the arc of the curved treadmill. Furthermore, this could indicate a greater stride length on the curved treadmill. Difference in knee motion was observed during the swing phase at the two highest walking speeds where the curved treadmill walking had greater knee flexion compared to the flat treadmill. This could be a compensating mechanism to the increased hip flexion prior and post heel strike. The difference in ankle joint motion did not affect the ankle joint range of motion. However, differences between treadmills were observed during the contact phase which could be explained by the curved surface of the treadmill not allowing the plantar flexion of the ankle during the initial part of the contact phase. The differences in joint angles between the two treadmills were not substantially greater than the differences between the walking speeds, indicating that change in speed induced changes in movement pattern more or less equal to those occurring when changing treadmill. The effect size calculations of hip and knee joint angle range of motion indicate large effect of the walking condition. Thus, while the SPM analysis revealed phase dependent differences in the movement patterns corresponding to changes observed with changes in walking speed, the effect size of the overall movement indicated considerable differences.

From an exercise perspective, the results of the present study would seem to be support the use of the curved treadmill in rehabilitation. The vertical displacement of the CoM is a reflection of the ground reaction force and a reduced displacement on the curved treadmill would be expected to be accompanied by a reduction in loading and unloading fluctuations. In addition, it could be speculated that the way the foot is ‘caught’ by the curved treadmill belt instead of a ‘fall’ on the flat treadmill could also potentially reduce the impact force applied to the foot. This, combined with the increased oxygen uptake, would seem to promote a more effective exercise mode on the curved treadmill with a potentially reduced lower limb joint loading compared to a traditional flat treadmill. Future research should investigate this potential advantageous aspect of curved treadmill use.

Increased oxygen uptake and heart rate have previously been measured during walking and running on a non-motorized and self-propelled curved treadmill, similar to the one used in the present study, as compared to a traditional motorized flat treadmill^30^. These observations were confirmed by the present study. However, it is possible that the increased oxygen uptake on the curved treadmill could be due to friction loss and the need to propel the treadmill belt. The results in the supplementary materials indicated that the use of a non-motorized curved treadmill did not bias the results. The differences in the vertical displacement of the CoM and in the oxygen uptake between the non-motorized curved treadmill and the flat treadmill were also observed when comparing the motorized curved treadmill and the flat treadmill. Furthermore, the similarities in stride characteristics and joint angle kinematics between the two treadmills in this study were also observed in the secondary study presented in the supplementary material.

It is worth mentioning that the used method to assess the vertical displacement of the CoM has been shown to slightly overestimate the result compared to a whole-body kinematic models and ground reaction force recordings^11,31^. However, this should be considered as a systematic inaccuracy for the results obtained on both treadmills. Thus, this should not change the interpretations of the results, but direct comparisons of results with our studies should be done with caution. Furthermore, the study by Gard and colleagues^11^ stated that the sacral marker is a reasonably good estimate of the vertical displacement of the CoM at walking speeds below 1.4 m/s. In the present study, the fastest walking speed was only slightly above this.

The present study estimated the vertical displacement of the center of mass from the vertical displacement of the sacrum marker in the global coordinate system. Alternatively, it could be suggested that the vertical displacement of the sacrum marker should be calculated relative to the curved surface of the treadmill belt. While this methodological approach might be appropriate in other experimental setting, we believe that the design of the curved treadmill makes it comparable to the flat treadmill in terms of conservation and exchange of mechanical energy which in our opinion justify the used methodology. We aim at challenging the notion by Saunders and colleagues^1^ that reducing the cyclic lift of the CoM is associated with a reduction in energy expenditure. Thus, our methodological approach indirectly addressed the cyclic exchange of potential energy within each stride by quantify the vertical displacement of the CoM in the global coordinate system. The methodological choice relies on the assumption that walking on the curved treadmill exhibits the same energy conserving exchange of potential and kinetic energy as during flat treadmill walking. Thus, the inverted pendulum model of level walking suggests that when assuming perfect energy conservation, walking does not cost additional energy once initiated due to the cycling exchange of energy forms. A similar model (bipedal with stiff legs) and similar assumption of energy conservation applied to the curved treadmill would mean that once initiated the leg motion (which in this case mimics a regular pendulum behavior) will swing back and forth along the curved belt exchanging energy without the loss of energy. In theory, this means that if a mass is placed on the sloped front part of the curved treadmill belt, the belt will start moving; the mass will be moved backwards and upwards until it reaches the same height as it was initially placed after which it then will return to the starting point. This is in contrast to walking on an inclined treadmill or a stairmill where there is no conservation of the mechanical energy within each stride. The methodological advantage of the curved treadmill is two-sided. First, it preserves energy with a cyclic exchange equal to that of level walking, and secondly, it enables a separation of the vertical displacement where a part of the displacement takes place at the distal end of the pendulum and the remaining part takes place at the proximal part. Since we assume energy equilibrium in the distal part, we believe that the vertical displacement of the proximal part in the global coordination system well represents ‘*…the summation or end result of all forces and motions acting upon and concerned with the translation of the body from one point to another during locomotion*.’, as stated by Saunders and colleagues (pp. 544).

Additionally, the measurement of vertical displacement relative to the treadmill surface may give a more accurate measurement of absolute CoM height and potentially negate the impact of possible differences in treadmill surface deflection as compared to the current study’s method of measuring CoM relative to the global coordinate system. The use of the treadmill surface, rather than the global coordinate system, would either not present any benefit over the global coordinate system with regards to displacement (if the height is calculated relative to a static treadmill surface height), or introduce increased noise and inaccuracy from measuring the difference between two markers that are being subjected to impact vibration. The vibration noise would be most amplified by a marker on the surface bed or the foot where vibrations are greater than a more proximal portion of the body. Furthermore, this study included a mix of female and male, and while female have been reported to have reduced vertical displacement of CoM^32^, this study utilized a repeated measures design to eliminate such concern. If the study would have restricted to only males, it would be expected to see the CoM displacement differences further exaggerated given males inclination for increased displacement.

The present study observed an increased oxygen uptake during walking on a curved treadmill which reduced the vertical displacement of the CoM compared to a flat treadmill. This reduction in vertical displacement of the CoM was not achieved by a deliberate change in the gait pattern and only accompanied by minor changes in the joint kinematics. However, it is unknown if and how the muscle activation pattern is affected by the curved treadmill. Future studies should investigate this next logical step.

## Conclusion

The present study supports the growing literature questioning the underlying purpose of the six determinants of gait proposed by Saunders and colleagues^1^. However, the uniqueness of our study is the novel experimental set-up used where we sought to determine the effect of reduced vertical displacement of the CoM on the oxygen uptake and walking economy without deliberately altering the natural gait pattern. We utilized two treadmills, curved and flat, to manipulate the vertical displacement of the body’s CoM without having individuals consciously alter their gait. We also utilized different walking speeds (0.67 m/s, 1.12 m/s and 1.56 m/s) to enhance the generalizability of our results by identifying the effect that speed may have on our outcomes. We found that decreasing the vertical displacement of the CoM using a curved treadmill was not accompanied by a reduction in oxygen uptake and walking economy. In contrast, the oxygen uptake increased during walking with reduced vertical displacement of the CoM. The use of the curved treadmill introduced phase dependent significant changes in the lower limb movement pattern.

## Electronic supplementary material

Supplementary information
