# Supplementary material for: Reduced vertical displacement of the center of mass is not accompanied by reduced oxygen uptake during walking
Source: Sci Rep. 2017 Dec 7;7:17182. doi: 10.1038/s41598-017-17532-6 (PMC5719393; doi:10.1038/s41598-017-17532-6)
Supplement: Supplementary file 1 — Supplementary information [file 41598_2017_17532_MOESM1_ESM.pdf]

SUPPLEMENTARY MATERIAL

Title:

Reduced vertical displacement of the center of mass is not accompanied by reduced oxygen uptake during walking

Authors:

Wurdeman, SR.[1,2], Raffalt, PC.[3,4] and Stergiou, N.[2,5]

Affiliations:

1 Department of Clinical and Scientific Affairs, Hanger Clinic, 11155 S. Main St., Houston, TX 77025, USA

2 Department of Biomechanics and Center for Research in Human Movement Variability, University of Nebraska at Omaha, 6160 University Drive, Omaha, NE 68182-0860, USA.

3 Julius Wolff Institute for Biomechanics and Musculoskeletal Regeneration, Charité – Universitätsmedizin Berlin, Augustenburger Platz 1, 13353 Berlin, Germany.

4 Department of Biomedical Sciences, University of Copenhagen, Blegdamsvej 3, 2200, Copenhagen N, Denmark.

5 College of Public Health, 984355 University of Nebraska Medical Center, Omaha, NE 68198-4355, USA.

Corresponding Author:

Nicholas Stergiou, PhD

Department of Biomechanics and Center for Research in Human Movement Variability

University of Nebraska at Omaha

6160 University Drive

Omaha, NE 68182-0860, USA.

Email: nstergiou@unomaha.edu

Phone: 402-554-3247

The purpose of the present supplementary material was to investigate any potential bias of the curved treadmill being non-motorized and compared to the motorized flat treadmill. Five subjects (2 males, 3 females) with a mean (SD) age of 23.8 (6.30) years, body mass of 67.85 (14.44) kg and height of 170.34 (6.83) cm walked on both a motorized curved treadmill and a motorized flat treadmill. All participants were informed of the experimental conditions and gave their written consent to participate in the study. The study was approved by the Institutional Review Board of the University of Nebraska Medical Center, and the study was carried out in accordance with the approved guidelines.

Similar experimental setup was used as during the main experiment, however, the subjects only walked at 1.12 m/s. Stride characteristics (stride time, contact and swing phase), vertical displacement of the centre of mass (CoM), hip, knee and ankle joint angles in the sagittal plane and the oxygen uptake were extracted for each subject and averaged across subjects. Due to the low number of included subjects, no statistical analysis was performed.

For all subjects the vertical displacement of CoM was higher during walking on the motorized curved treadmill compared to the flat treadmill (figure S1). The group average values and standard deviation corresponded relative well to the value of the main experiment. However, the vertical displacement during curved treadmill walking appeared to be slightly higher compared to the non-motorized curved treadmill.

The oxygen uptake data showed the same pattern as that presented in the main study (figure S2). During motorized curved treadmill walking the oxygen uptake was higher compared to walking on the flat treadmill. The values lied well within the range of the data presented in the main study.

The stride characteristics did not appear to differ between the two treadmills confirming the observations of the main study (table S1). The values were in general similar, however, the stride time during walking on the motorized curved treadmill appeared shorter compared to during walking on the non-motorized curved treadmill.

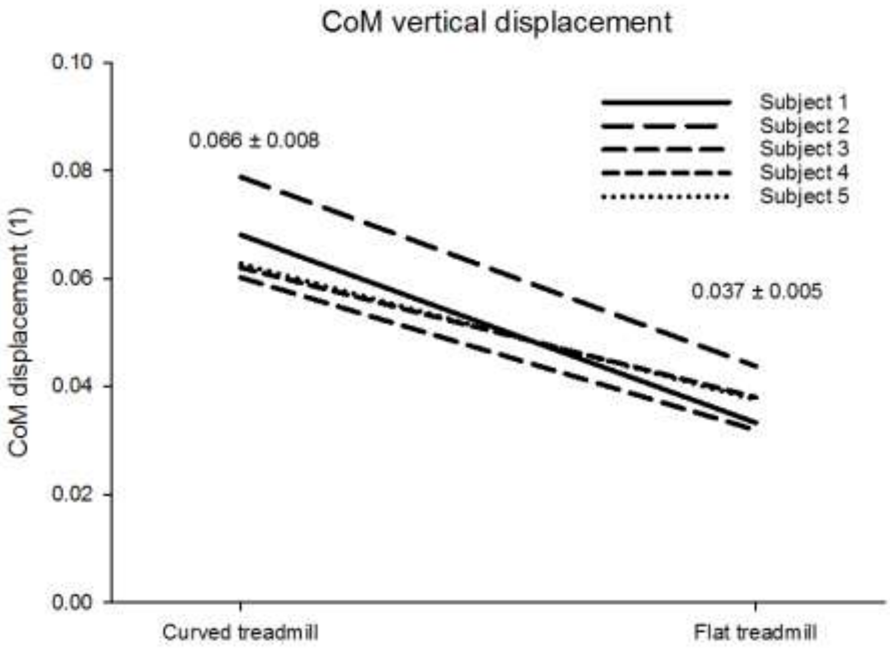

Figure S1: Vertical CoM displacement for all five subjects during 1.12 m/s walking at the curved treadmill and the flat treadmill. Group mean and standard deviation are stated.

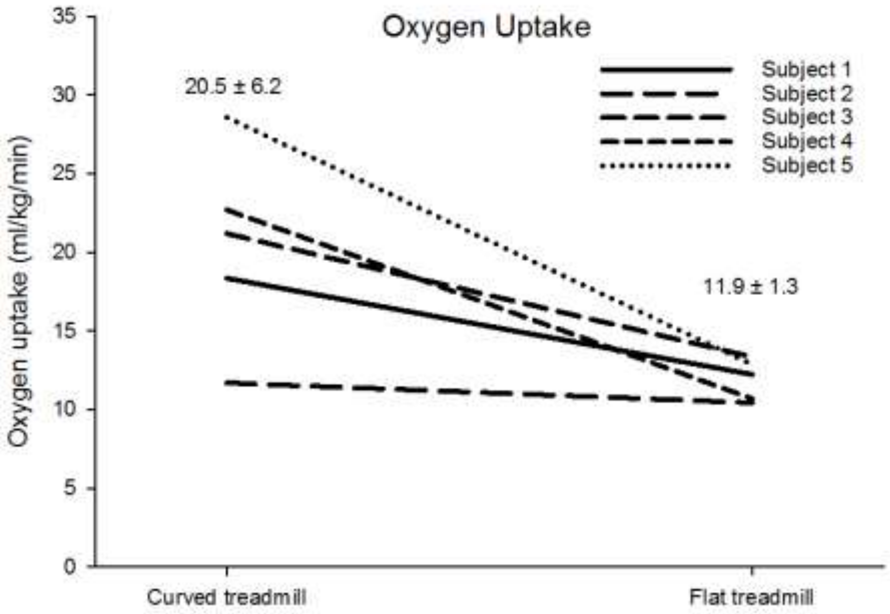

Figure S2: Oxygen uptake (top graph) and walking economy (bottom graph) for all five subjects during 1.12 m/s walking at the curved treadmill and the flat treadmill. Group mean and standard deviation are stated.

Table S1: Stride characteristics (mean ± SD) during curved and flat treadmill walking at 1.12m/s.

	Curved treadmill	Flat treadmill
Right ST (s)	1.04 ± 0.06	1.12 ± 0.05
Left ST (s)	1.04 ± 0.06	1.12 ± 0.05
Right CT (s)	0.63 ± 0.04	0.69 ± 0.03
Left CT (s)	0.63 ± 0.04	0.70 ± 0.03
Right SWT (s)	0.41 ± 0.02	0.42 ± 0.01
Left SWT (s)	0.41 ± 0.02	0.42 ± 0.01

ST: stride time, CT: contract time, SWT: swing time.

The joint angles confirmed the observations in the main study. The range of motion for the three joints was almost identical to that reported in the main study (table S2). Furthermore, limited differences appeared between the motorized curved treadmill and the flat treadmill (figure S3).

Table S2: Range of motion (mean ± SD) of the right hip, knee and ankle joint angle during curved and flat treadmill walking at 1.12m/s.

	Curved treadmill	Flat treadmill
Hip (°)	40.1 ± 6.0	31.9 ± 2.6
Knee (°)	65.1 ± 5.4	59.3 ± 4.6
Ankle (°)	25.2 ± 8.0	24.8 ± 3.3

The present supplementary material does not indicate any bias with respect to vertical displacement of the CoM, oxygen uptake, stride characteristics and joint angles due to the flat treadmill being motorized and the curved treadmill not being. The differences and similarities observed between the motorized curved treadmill and the motorized flat treadmill resembled the differences and similarities observed for the non-motorized curved treadmill and the flat treadmill in the main study.

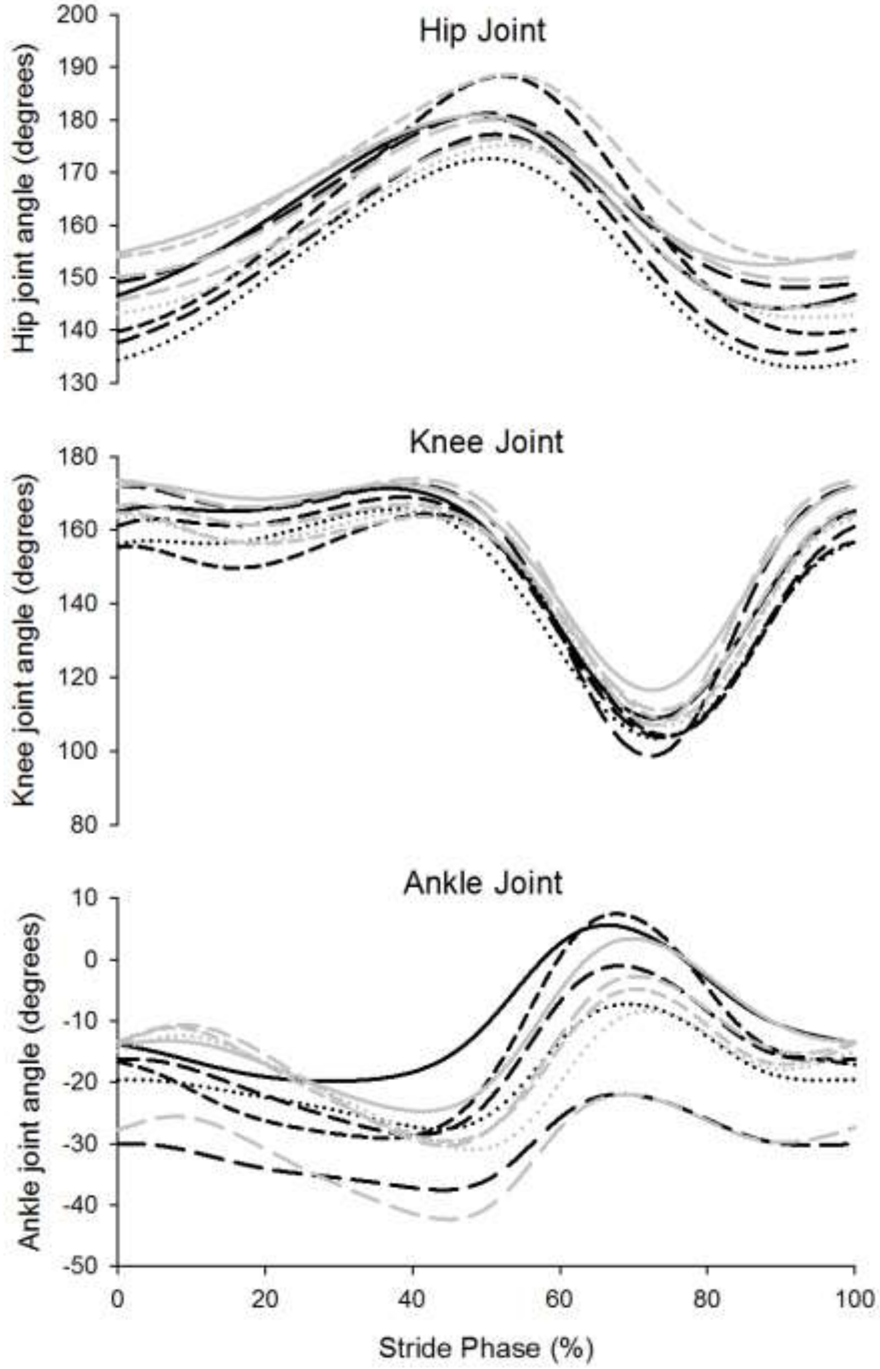

Figure S3: Joint angles for the hip joint (top graph), knee joint (middle graph), ankle joint (bottom graph) for each subject during walking on the motorized curved treadmill (black lines) and the flat treadmill (grey lines) at 1.12 m/s.
